# PReS-FINAL-2303: Exploring potential differences in demographics, family history and disease characteristics in JSLE patients with different age of onset

**DOI:** 10.1186/1546-0096-11-S2-P293

**Published:** 2013-12-05

**Authors:** N Groot, AM Midgley, L Watson, C Roberts, MW Beresford

**Affiliations:** 1 Institute of Child Health, University of Liverpool, Alder Hey Children's NHS Foundation Trust Hospital, Liverpool, UK

## Introduction

Juvenile-onset SLE (JSLE) is a severe auto-immune disease that can occur in children at any age. Variations regarding the extent of organ involvement, disease activity and damage are found in different age categories. Factors that may contribute to an earlier age of onset are gender, ethnicity and positive family history of SLE. It has been shown that JSLE has a more aggressive disease course compared to adult onset SLE. Correspondingly, it seems that disease severity in younger JSLE patients increases with decreasing age ^1,2,4^. However, there is some conflicting evidence.

## Objectives

To determine the differences in gender, ethnicity and family history of SLE in JSLE patients with age of onset <10 years and >10 years respectively, and to characterize differences in disease characteristics (namely activity, damage, organ involvement) between these groups.

## Methods

Data on patient demographics, family history and disease activity (BILAG) and damage (SLICC) at diagnosis and last follow-up visit were collected from the UK JSLE Cohort Study database. Patients were divided into two age categories based on age of onset (≤10 or >10 years) and were analyzed. BILAG scores of a subset of patients (n = 24) who were followed for five years were analyzed, to evaluate influence of disease duration.

## Results

A total of 313 patients with JSLE, diagnosed using the ACR-criteria, were included in the analysis. Their data is summarized in the following table: The percentage of female patients was 80% in the <10 group and 84% in the >10 group (p = 0.423). However, the proportion of black children in the <10 group was higher (p = 0.073) and the proportion of Caucasian children in the <10 group was significantly lower (p = 0.047). Family history for SLE was not significantly different between the age categories (p = 0.121).

Disease activity at onset was higher in the older group but not significantly (p = 0.105) and no significant differences were found in organ systems involvement. This is in contrast with most literature. Referral time might be a factor causing this and would be an interesting factor to study in this population. Disease activity and damage at follow up (disease duration <10: 4 ± 4 years, >10: 2 ± 2 years) were low in both groups, irrespective of disease duration. Five years after disease onset, disease activity in the <10 group (n = 15) was 1 ± 3, in the >10 group (n = 9) it was 4 ± 5 (p = 0.290). This indicates that for most patients in this cohort, their disease is probably managed well.

## Conclusion

In contrast to other published cohorts, there were no striking differences in demographics, family history or disease characteristics in this cohort between children diagnosed before or after the age of 10 years old.

## Disclosure of interest

None declared.

**Table 1:** 

Age at Diagnosis	<10 yrs, n = 92	>10 yrs, n = 221
Ethnicity: Black Asian Caucasian	15% 27% 46%	7% 28% 53%
Family history SLE	16%	11%
BILAG at onset **(median ± IQR)**	5.5 ± 12	8.0 ± 11
BILAG at last visit **(median ± IQR)**	2.00 ± 3	2.00 ± 3

